# Environmental-friendly Eco-labeling Matters: Evidences From an ERPs Study

**DOI:** 10.3389/fnhum.2018.00417

**Published:** 2018-10-12

**Authors:** Jia Jin, Xiaodong Dou, Liang Meng, Haihong Yu

**Affiliations:** ^1^Business School, Ningbo University, Ningbo, China; ^2^Academy of Neuroeconomics and Neuromanagement, Ningbo University, Ningbo, China; ^3^Law School, Ningbo University, Ningbo, China; ^4^School of Business and Management, Shanghai International Studies University, Shanghai, China; ^5^Faculty of Maritime and Transportation, Ningbo University, Ningbo, China

**Keywords:** eco-labeling, ERPs, P2, N2, emotion, purchase intention

## Abstract

Nowadays, the international community is becoming increasingly concerned about the sustainable utilization of natural resources. In order to protect the environment and reward sustainable practices, eco-labeling that signifies the environmental friendliness of the labeled food is already widely promoted in many regions around the world. Thus, it is of great importance for researchers to study consumers’ attitudes toward eco-labeled food as food is supposed to satisfy consumers’ needs. This study employed the event-related potentials (ERPs) approach to investigate consumers’ attitudes toward eco-labeled food by comparing their neural processing of visual stimuli depicting eco-labeled and non-labeled food. Our results showed that behaviorally, participants preferred to buy eco-labeled food rather than non-labeled one. At the neural level, we observed markedly smaller P2 and N2 amplitudes when pictures of eco-labeled food were presented. Furthermore, we also found that amplitudes of P2 were negatively correlated with participants’ purchase intention. Therefore, our current findings suggest that, while the environmental-friendly eco-labeling was not to one’s own interests, it might still be evocative, which induce consumers’ positive emotion, bring less cognitive conflict to the purchase decision-making and then result in a greater purchasing intention. This effect might be the result of the delivered value of social desirability.

## Introduction

Currently, environmental protection has become an important issue all over the world. As a result, the sustainable utilization of natural resources has claimed widespread attention from both researchers and practitioners. A series of policies and regulations have already been formulated for the protection of the environment and natural resources. Among the implemented policies, one of the most important and effective policies is the set-up of standards for environmental-friendly labels, which will help rectify order of the food market by recognizing and rewarding sustainable practices and influencing the choices people make when buying food products. Examples of existing programs and labels include Friends of the Sea, KRAV (Sweden), Label Rouge (France), Marine Eco-Label Japan, and Marine Stewardship Council’s (MSC’s) label.

As food products are supposed to satisfy consumers’ needs, it is interesting and significant to promote eco-labeling by studying consumers’ preference toward eco-labeled food. Previous studies have shown that consumers generally hold positive attitudes toward eco-labeled products. For example, people tend to prefer the taste of a cup of coffee they believe to be “eco-friendly” over another cup that is believed to be “conventional,” even if the two cups of coffee are actually identical (Sörqvist et al., 2013). Similar effects have been found across a range of products, including bread (Annett et al., 2008), bananas (Sörqvist et al., 2015), seafood (Wessells et al., 1999; Johnston et al., 2001) and clean energy (Nilsson et al., 2014). For instance, Johnston et al. (2001) compared consumers’ preferences for eco-labeled seafood in the United States and Norway. They found that consumers preferred eco-labeled seafood in both countries. In China, Xu et al. (2012) found that Chinese consumers considered the seafood label as a more important information source compared with their own consumption experience, and they were willing to pay more for the eco-labeled seafood in order to protect societal benefits (Xu et al., 2012). While most of these studies examined consumers’ preferences toward eco-labeled food on the behavioral level, few studies tried to explore the emotional experience and cognitive process underlying consumers’ explicit preferences. As researchers suggested that capture of the emotional experience of consumers would help marketing professionals better understand consumers’ preferences and then boost sales (Gountas and Gountas, 2007), this study aimed to examine the emotional experience and cognitive process underlying one’s preferences for eco-labeled vs. non-labeled food. Specifically, we intended to explore whether there are discrepancies in both consumers’ purchase intention and corresponding brain activities when they are exposed to eco-labeled vs. non-labeled food.

In recent years, with the development of non-invasive technologies, researchers began to measure one’s cognitive and affective processes adopting neuroscientific methods such as event-related potentials (ERPs). These neuroscientific methods are believed to provide information that is not obtainable through conventional marketing method such as scales and interviews (Boksem and Smidts, 2015). Indeed, previous Consumer Neuroscience studies have made substantial contributions to the understanding of consumers’ decision-making by investigating their cognitive and affective processes. For example, Wang et al. (2012) investigated consumers’ affective responses to pendants by using ERPs. They reported that less beautiful pendants elicited larger P2 amplitudes than more beautiful ones. As more positive emotions were reported to give rise to a less pronounced P2, this finding suggested that beautiful pendants might induce more positive emotions (Wang et al., 2012). Thus, this study confirmed the involvement of the human emotional system in consumers’ decision-making process.

When it comes to neuroscientific investigations of consumers’ evaluation of food label, a pioneer study employed the functional magnetic resonance imaging (fMRI) technique to examine consumers’ evaluation of the organic labeling, which found increased activities in the ventral striatum for foods labeled as “organic” in comparison to conventionally labeled food (Linder et al., 2010). Some follow-up studies have examined nutrition labels (Enax et al., 2015), health labels (Grabenhorst et al., 2013), as well as controversial food label (Lusk et al., 2015). It is worth noting that all these existing studies examined consumers’ attitude toward self-beneficial label, which highlight the benefit for the consumers themselves. However, there also exist other kinds of labeling, which highlight that the main beneficiary of support is some other individual or organization. According to previous studies in marketing research (Fisher et al., 2008; White and Peloza, 2009), we refer this kind of labeling as “social-benefit.”

Since previous neuroscience studies suggested that social rewards would activate the same reward circuitry (the striatum) as monetary rewards (Izuma et al., 2008), in this study we would like to examine whether the social-beneficial food label would also activate the same reward circuitry as the self-beneficial label. Compared with fMRI which has a high spatial resolution and a low temporal resolution, the ERPs technique is more affordable and can provide a high temporal resolution, which can reveal timing of brain activities (Friedman and Jonson, 2000). Therefore, in this study ERPs was adopted to examine consumers’ evaluation of environmental-beneficial eco-labeling and to compare the neural differences between one’s processing of self- and others-beneficial food labels.

Previous studies consistently suggested that ERPs is a valuable technique to illuminate consumers’ decision-making process across multiple marketing-related domains, particularly those underlying emotions and preferences (Yoon et al., 2012; Smidts et al., 2014; Camerer and Yoon, 2015). Specifically, early ERP components, which refer to those appear at the first 300 ms after stimulus onset, were reported to reflect initial sensory encoding of emotionally significant stimuli (Junghöfer et al., 2001; Schupp et al., 2007). P2 is one of the most commonly examined early ERP components (e.g., Wang et al., 2012). Numerous ERPs studies have reported that the positive-going component P2 with a peak latency from 100 ms to 200 ms was sensitive to the emotional valence of presented stimuli (Paulmann and Kotz, 2008; Lai and Huettig, 2016). Furthermore, existing studies found that P2 typically showed a higher amplitude in response to negative stimuli than positive ones (Carretié et al., 2001; Huang and Luo, 2006; Lai and Huettig, 2016). That is, the P2 component was found to be modulated by the valence of one’s emotion in response to affective stimuli.

N2 is another frequently studied ERP component in Consumer Neuroscience literatures. It typically peaks at approximately 250–350 ms after the onset of a stimulus (Folstein and Van Petten, 2008). Previous studies suggested that the N2 component is related to the cognitive control or conflict monitoring (Folstein and Van Petten, 2008). The typical N2 can be elicited by the go/no go paradigm and reaches its maximum in frontal areas. For example, in Eimer’s (1993) work, the participants were asked to respond to a specific letter (go stimulus) but not to another one (non-go stimulus). Their results showed that a larger N2 amplitude was elicited by the non-go stimulus than the go stimulus (Eimer, 1993). In Consumer Neuroscience domain, the N2 was also found to reflect the cognitive control or conflict monitoring while evaluating the marketing-related stimuli (Ma et al., 2010; Jin et al., 2017; Shang et al., 2017). For example, Shang et al.’s (2017) work found that a larger N2 amplitude was induced by perception of a social risk in contrast with the control condition during the evaluation of a product. This finding was explained that N2 may reflect the cognitive control or conflict monitoring as the participants have to regulate the cognitive conflict between an inherent desire to purchase the item and the discordant information they obtained from social interactions (Shang et al., 2017). Furthermore, in a more recent study, Jin et al. (2017) found the larger N2 amplitude can also be induced by negatively framed market information compared with positively framed one. Based on existing findings, we hypothesized that negative marketing stimuli will bring a greater cognitive control or enhanced conflict monitoring during consumption decision-making and thus elicit a larger N2 (negative polarity) compared with positive marketing related stimuli.

As has been introduced above, in the current study the ERPs was adopted to investigate consumers’ attitudes toward eco-labeled food at the brain level. According to the aforementioned findings, we hypothesized that in the current study, eco-labeled and non-labeled seafood pictures may induce different brain activities, which would be manifested in discrepancies in P2 and N2 amplitudes. As the P2 can reflect the emotional valence of stimuli, we posited that eco-labeled seafood may elicit a smaller P2 amplitude than the non-labeled one, as participants would generally have more positive feelings toward eco-labeled seafood. In addition, as negative marketing related stimuli would elicit a larger N2 compared with positive marketing related stimuli, we also predicted the non-labeled seafood to elicit a larger N2 than the eco-labeled one.

## Materials and Methods

### Participants

Twenty-one (M = 13, F = 8) right-handed healthy undergraduates and graduates from Ningbo University were recruited to participate in the current experiment. Their ages ranged from 19 to 25, with a mean age of 20.94 (SD = 1.39). All of the participants were native Chinese speakers without any history of neurological disorder or mental diseases. Their visual acuity was normal or corrected-to-normal. This study was carried out in accordance with the recommendations of Academy of Neuroeconomics and Neuromanagement at Ningbo University. The protocol was approved by the Academy of Neuroeconomics and Neuromanagement at Ningbo University. All subjects gave written informed consent in accordance with the Declaration of Helsinki. Data from two male participants were discarded because of excessive artifacts during electroencephalogram (EEG) recordings. Thus, data from 19 valid participants entered into the final analysis.

### Materials

This experiment has two experimental conditions (eco-labeled vs. non-labeled seafood), and there are 80 trials in each condition. Thus, the whole experiment involved 160 stimuli (80 seafood × 2 label categories). The 80 seafood pictures depict 20 fishes (e.g., *Pseudosciaena crocea*, pomfret, groupers, sierras, and squids), 20 shellfishes (e.g., oysters, razor clam, small clam, sea scallop and arctic shellfish), 20 crabs (e.g., shuttle crab, green crab, king crab, Dungeness crab, and tourteau) and 20 shrimps (e.g., prawn, lobster, squilla, greasyback shrimp, and *Penaeus orientolis*). To be standardized, all pictures were downloaded from the Internet and edited by Photoshop 7.0 (Adobe Systems Incorporated, San Jose, CA, USA).

The selected eco-labeling adopts the MSC label, which is certified by the most prominent eco-labeling certifier, the MSC. This Council is an international non-profit organization established to address the problem of unsustainable fishing and to safeguard seafood resources for the future. The blue MSC label makes it easier for everyone to find seafood that has been caught by fisheries that care for the environment. Because MSC is a label for seafood, only seafood pictures were selected to prepare the stimuli.

The standardization process takes the following steps: (1) the seafood image was tailored and processed to the size of 300*150 pixels and was located in a 270*360 pixel black background; and (2) consistent with Linder et al.’s (2010) work, each stimulus contained a food picture with a label. Seafood pictures were shown along with the original MSC label in the labeled condition, while the same food pictures were shown with an artificially created logo indicating production of that food in a conventional manner in the non-labeled condition. In other words, the food pictures used were identical in these two conditions, and the only difference lies in the label. The label was located in the top-right corner of the stimuli, the size of which was 70*100 pixel.

A pretest was conducted to ensure that subjects in the formal experiment can recognize most of the seafood demonstrated in the picture. Only when a participant candidate could recognize at least 75% of the seafood that would appear in the experiment could they pass the pretest and move on to the formal experimental. All the stimuli were randomly and evenly divided into four blocks in the formal experiment.

### Procedure

Participants were asked to sit in a sound-attenuated room 100 cm away from a computer-controlled monitor, on which the stimuli were presented. They were provided with a keypad to report their purchase intention of each product through a 4-point scale. Before the experiment started, the participant received a brochure introducing the meaning of the MSC label, including its certification authority, mission and so on.

**Figure 1:**
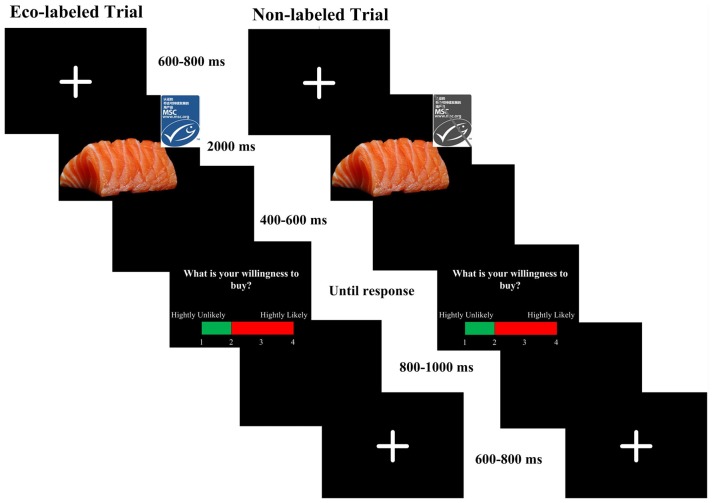
Experimental task: participants were instructed to report their purchase intention toward two kinds of food (eco-labeled and non-labeled) on a 4-point scale. Electroencephalograms (EEGs) were recorded from the subjects throughout the experiment.

As shown in Figure 1, each trial began with a fixation cross against a black background for 400–600 ms, which was followed by a blank screen lasting for 500 ms. Afterwards, a picture with a specific seafood and a label appeared for 2,000 ms. Then, the participants were asked to rate their purchase intention for the current item on a 4-point scale (1 means the lowest purchase intention, and 4 means the highest purchase intention). Stimuli, recording triggers and response data were presented and recorded using E-Prime 2.0 (Psychology Software Tools, Pittsburgh, PA, USA). The participants were asked to minimize blinks, eye movements, and muscle movements during the whole experiment. The formal experiment started after 10 practice trials.

### Electroencephalogram (EEG) Recording and Analysis

EEGs were recorded with a cap containing 64 Ag/AgCl electrodes and a Neuroscan Synamp2 Amplifier (Scan 4.5, Neurosoft Labs, Inc). Its sampling rate was 500 Hz, and channel data were online band-pass-filtered from 0.05 Hz to 70 Hz. The experiment started only when electrode impedances were reduced to below 5 kΩ. A cephalic (forehead) location between FPz and Fz was used as the ground, and the left mastoid was used for reference. Electrooculograms (EOGs) were recorded from electrodes placed at 10 mm from the lateral canthi of both eyes (horizontal EOG) and above and below the left eye (vertical EOG), and EOG artifacts were off-line corrected for all subjects using the method proposed by Semlitsch et al. (1986).

EEG data were off-line transformed based on the average of the left and right mastoid references. EEG recordings were digitally filtered with a low-pass filter at 30 Hz (24 dB/Octave). For ERP analyses, the data were segmented for the epoch from 200 ms before the onset of stimulus on the video monitor to 800 ms after its onset, with the first 200 ms pre-target interval as a baseline. Trials containing amplifier clippings, bursts of electromyography activity, or peak-to-peak deflections exceeding ±100 μV were excluded. For each participant, EEG recordings were averaged for the two experimental conditions (eco-labeled vs. non-labeled) over each recording site.

The time window of 160–220 ms was chosen for the analysis of P2 based on visual observation and the guideline proposed by Picton et al. (2000). Ten electrodes (F3, F1, Fz, F2, F4, FC3, FC1, FCz, FC2 and FC4) in the frontal-central area were included into the statistical analysis. A 2 (Label: eco-labeled vs. non-labeled) × 10 (electrodes) ANOVA was conducted for the P2 analysis. Spearman correlation analysis was conducted between the P2 amplitude and participants’ purchasing intention.

When it comes to the N2 component, from visual inspection of the grand averaged waveforms, it occurred to us that the waveform of N2 (300–400 ms) is superimposed on its preceding positive deflection (160–220 ms). Therefore, we used the peak-to-peak measure instead of the mean amplitude approach when analyzing the N2. As was suggested by Picton et al. (2000), the use of a peak-to-peak measure is justified in the following conditions: (1) a peak is superimposed on a slower wave or (2) an adjacent peak-trough ensemble is considered to reflect the same functional process. Within each averaged waveform, the amplitudes of the distinct peaks of the two conditions were measured as follows: first, a positive peak was identified as the most positive peak within 160–220 ms after stimulus onset. Second, a negative peak (N2) was defined as the most negative peak observed within 300–400 ms after stimulus onset. The peak-to-peak amplitude of the N2 was obtained by subtracting amplitude of the positive peak amplitude from the negative peak. Then, a 2 (Label: eco-labeled vs. non-labeled) × 6 (electrode: F1, Fz, F2, FC1, FCz and FC2) ANOVA was performed for the N2 analysis.

The Greenhouse-Geisser (Greenhouse and Geisser, 1959) correction was applied for violation of the sphericity assumption in appropriate parts of the ANOVA (uncorrected *df*s were reported with ε and the corrected *p*-values). Effect sizes are provided as partial eta squared ($\eta_{\text{p}}^{2}$).

## Results

### Behavioral Results

**Figure 2:**
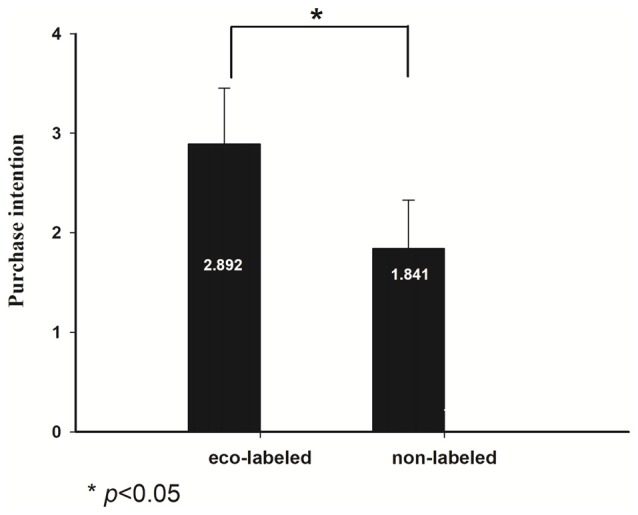
Behavioral results of the purchasing intention: subjects’ purchase intentions of the two kinds of food (eco-labeled and non-labeled) are provided.

Behavioral data was shown in Figure 2. A pairwise *t*-test was conducted between purchase intention of eco-labeled and non-labeled food, which showed a significant main effect (*t*_(1,18)_ = 6.730, *p* < 0.001). This finding indicated that subjects had a greater willingness to buy eco-labeled food (M = 2.862, SD = 0.554) compared with non-labeled food (M = 1.780, SD = 0.523).

### ERP Results

#### P2 Analysis

**Figure 3:**
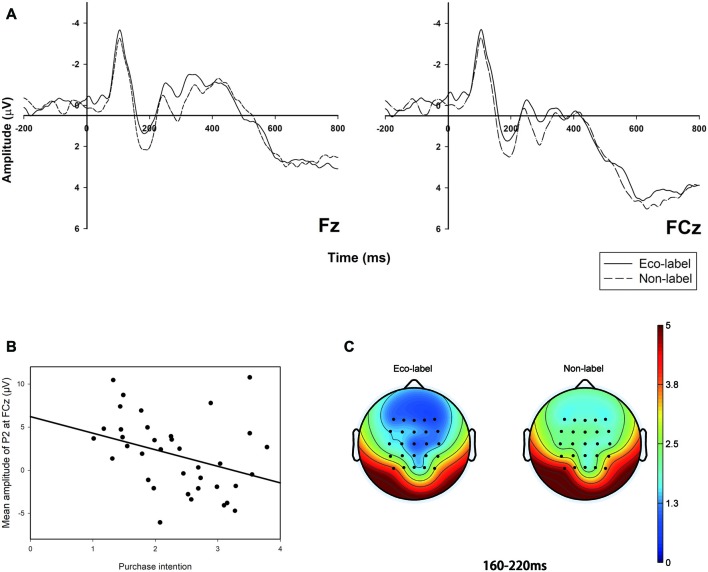
P2 Grand-averaged event-related potential (ERP) waveforms in the frontal region with two electrodes, linear correlation between the amplitude of certain ERP components and behavioral results as well as the brain topography: **(A)** the comparison between eco-labeled and non-labeled food conditions in representative electrodes (Fz and FCz); the solid line represents eco-labeled food, whereas the dashed line represents the non-labeled food; **(B)** linear correlation between the amplitude of P2 and the participants’ purchase intention; **(C)** the brain topography of the two conditions at the P2 time window of 160–220 ms.

The two-way 2 (label) × 10 (electrodes) ANOVA for P2 amplitude in the time window of 160–220 ms suggested a significant main effect of label (*F*_(1,18)_ = 8.632, *p* = 0.009, $\eta_{\text{p}}^{2}$ = 0.324). As shown in Figure 3A the eco-labeled condition (M = 1.178 μV, SE = 0.911) elicited a smaller P2 amplitude (positive polarity: a larger voltage value means a larger amplitude) than the non-labeled condition (M = 1.923 μV, SE = 0.898).

We calculated the Spearman correlation between P2 amplitude on FCz and participants’ purchase intention. Since the amplitude of P2 reached its peak on FCz in both conditions, we take FCz as an example. As shown in Figure 3B, the P2 amplitude in FCz was negatively correlated with participants’ purchase intention of the seafood (*r* = −0.416, *p* = 0.009), which suggested that a larger P2 amplitude will be observed when participants have less willingness to buy the product. Magnitudes of all the 10 chosen electrodes as well as the average amplitude were significantly correlated with the purchase intention as well as shown in Table 1. The brain topography was shown in Figure 3C, which showed that the main difference between the two conditions was in the frontal part.

**Table 1:** Spearman correlation results between P2 amplitude and participants’ purchase intention.

	*R*^2^	*p*
F3	−0.424	0.008
F1	−0.390	0.016
Fz	−0.396	0.014
F2	−0.380	0.019
F4	−0.332	0.042
FC3	−0.498	0.001
FC1	−0.423	0.00
FCz	−0.416	0.009
FC2	−0.415	0.010
FC4	−0.438	0.006
Mean amplitudes	−0.407	0.011

#### N2 Analysis

**Figure 4:**
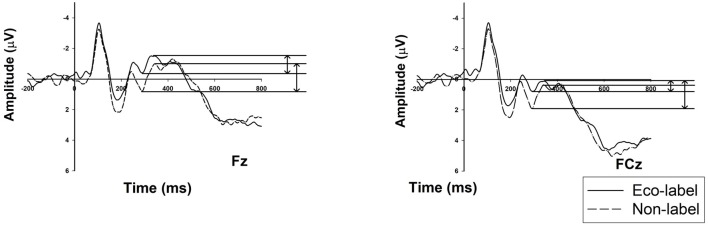
N2 Grand-averaged ERP waveforms in the frontal region with two electrodes: the comparison between eco-labeled and non-labeled food conditions in representative electrodes (Fz and FCz); the solid line represents eco-labeled food, whereas the dashed line represents the non-labeled food.

The results of the two-way 2 (label) × 6 (electrodes) ANOVA for N2 amplitude are shown in Figure 4, which suggested that the non-labeled condition (M = −4.157 μV, SE = 0.633) elicited a significantly larger N2 amplitude compared to the eco-labeled condition (M = −3.376 μV, SE = 0.448; *F*_(1,18)_ = 5.262, *p* = 0.034, $\eta_{\text{p}}^{2}$ = 0.226).

## Discussion

By adopting the ERPs approach, the present study explored neurocognitive processes associated with consumers’ attitude and emotion toward eco-labeled food. Behaviorally, participants’ purchase intention of eco-labeled food is significantly greater than non-labeled one. This finding is in accordance with the previous behavioral and empirical studies which suggested that participants preferred to buy eco-labeled food (e.g., Wessells et al., 1999; Xu et al., 2012).

A highlight of this study is that we explored consumers’ evaluation of environmental-beneficial eco-labeling and compared the neural differences between one’s processing of self- and others-beneficial food labels. Specifically, we observed a markedly smaller P2 when pictures of eco-labeled food were presented. We conjectured that it suggests that compared with non-labeled food, eco-labeled food would induce more positive emotions. Evidences from existing studies jointly provide rationale for our conjecture. First, as has been mentioned in the introduction, the P2 represents preliminary evaluation of the affective content of stimuli, and a decreased P2 amplitude is observed when displayed stimuli give rise to positive feelings (Carretié et al., 2001; Wang et al., 2012). Second, Previous studies on eco-labeling showed that it can successfully evoke one’s positive emotions (Atkinson and Rosenthal, 2014). Third, the current behavioral results showed that participants’ purchase intention of eco-labeled food is larger compared with non-labeled food. Subsequent analysis also showed that the P2 amplitude was negatively correlated with participants’ purchase intention in this study. According to previous studies, positive emotions to marketing stimuli are positively related with behavioral intentions (White and Yu, 2005; Jang and Namkung, 2009; Kim and Lennon, 2013). Last but not least, the current paradigm is adapted from Linder et al.’s (2010) work. In their study, the fMRI was adopted. Processing of organic-labeled food information was found to increase activities of the ventral striatum, which is responsible for emotional processing, compared with that of conventionally labeled food info (Davidson and Irwin, 1999; Linder et al., 2010). To conclude, as both social and monetary rewards were found to activate the same striatum area (Izuma et al., 2008), our findings may support the hypothesis that consumers’ preference can be reflected in brain activities, and specifically, the preferred eco-label can induce more positive emotions.

As a complementary finding, we also observed a significantly larger N2 in the non-labeled condition than in the eco-labeled one. As has been introduced in the introduction, N2 is a negative deflection which reflects the cognitive control or conflict monitoring brought by marketing stimuli. Therefore, the current results suggested that participants have to implement enhanced conflict monitoring while making the purchase intention of non-labeled food. This is consistent with the P2 and behavioral results, which suggested that eco-label food would induce more positive emotions and result in a higher purchase intention. Thus, we considered that consumers preferred the eco-labeled food emotionally and behaviorally and involved less cognitive conflicts.

Previous studies have reported similar results during the evaluation of organic-labeled food (Linder et al., 2010). However, the organic-labeled food pays more attention to the benefit of consumers themselves, while eco-labeled food focuses on its non-harm to the environment. Thus, as a complement to previous findings (Linder et al., 2010), our findings suggested that the environmental beneficial labeling can also induce consumers’ positive emotions and lead to positive behavioral preferences. Another highlight of this study is that, different from previous studies, the high temporal resolution technique of ERPs was adopted. Both of the two ERP components being examined are at the stage of early automatic processing, which reflect preliminary sensory encoding of stimuli. Thus, our findings suggest that environmental-friendly eco-labeling can elicit one’s positive emotions at early stage of cognitive processing.

Previous studies suggested that in addition to paying attention to one’s own interest, one’s thoughts and actions may also focus on activities that are evolutionary and adaptive, which contribute to the accumulation of enduring personal resources, such as development and maintenance of social relationships (Fredrickson, 1998; Fredrickson et al., 2008). Therefore, a potential mechanism underlying the observed effect in the current study might be social expectation, as to comply with the socially desirable act is beneficial to the development and maintenance of social relationships. Specifically, as behaving pro-environmentally is socially desirable and expected by members of the society as a whole (Milfont, 2009), the environmentally friendly behavior of eco-labeling can induce positive emotions and bring less cognitive conflict, which leads to the preference of environmentally beneficial eco-labeling.

We acknowledge some limitations in the current study. First, the sample size is relatively small. Only 19 valid participants were included in the final data analyses. Although the effect sizes of the current results are large enough according to previous studies, which stated that an effect size greater than 0.2 represents a large effect (Cohen, 1988), a greater sample size is always welcome to further verify the basic findings. Second, subjective preference for seafood was not measured in this study. When designing this experiment, we did not measure subjective preference for seafood, considering that this experiment has a within-subject design and the only difference between the two experimental conditions lies in the label. In other words, we examined the same participant’s attitude to both labeled and non-labeled seafood. If one has (or does not have) a preference for seafood, his/her attitudes toward labeled and non-labeled seafood may still vary. However, after repeated deliberation, we deem that different evaluations of the seafood may further influence one’s concern about the eco-label. This is what we neglected to consider when conducting this experiment, and follow-up studies that address our limitations are highly welcome.

To conclude, by employing the ERPs approach, the current study provided electrophysiological evidences for consumers’ preference for and positive emotions toward eco-labeled food. Specifically, we found the environmental-friendly eco-labeling to arouse consumers’ positive feelings and to bring less cognitive conflict, which were reflected in decreased P2 and N2 amplitudes, respectively. The current finding further suggests that, although the environmental-friendly eco-labeling was socially beneficial, which was not to the consumers’ own interests, it would still induce their positive emotions to the product and then result in a greater purchasing intention. The socially desirable theory was adopted to give a tentative explanation to this phenomenon. Additionally, this study shows the value of neuroscientific methods in revealing consumers’ (implicit) emotions as well as predicting their behavioral responses in studies of consumer behaviors.

## Author Contributions

JJ, XD, LM and HY conceived the idea; wrote the article. JJ, LM and HY prepared the experimental stimuli. JJ collected the data and ran the data analysis. XD supervised the project. All authors made intellectual contributions to this project and gave approval to the final version of the manuscript for submission.

## Conflict of Interest Statement

The authors declare that the research was conducted in the absence of any commercial or financial relationships that could be construed as a potential conflict of interest.
